# “We Are Here!” Oxygen Functional Groups in Carbons for Electrochemical Applications

**DOI:** 10.1021/acsomega.2c00639

**Published:** 2022-04-03

**Authors:** Mária Jerigová, Mateusz Odziomek, Nieves López-Salas

**Affiliations:** † Colloid Chemistry Department, Max Planck Institute of Colloids and Interfaces, Am Mühlenberg 1, 14476 Potsdam, Germany

## Abstract

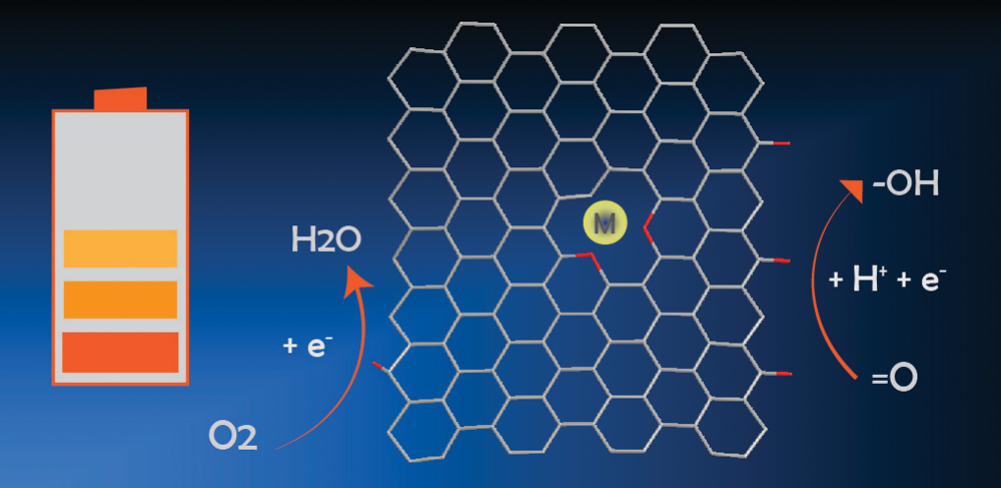

Heteroatom doping of carbon networks may introduce active functional groups on the surface of the material, induce electron density changes that alter the polarity of the carbon surface, promote the formation of binding sites for molecules or ions, or make the surface catalytically active for different reactions, among many other alterations. Thus, it is no surprise that heteroatom doping has become a well-established strategy to enhance the performance of carbon-based materials for applications ranging from water remediation and gas sorption to energy storage and conversion. Although oxygen functionalization is sometimes inevitable (i.e., many carbon precursors contain oxygen functionalities), its participation in carbon materials performance is often overlooked on behalf of other heteroatoms (mainly nitrogen). In this Mini-review, we summarize recent and relevant publications on the effect that oxygen functionalization has on carbonaceous materials performance in different electrochemical applications and some strategies to introduce such functionalization purposely. Our aim is to revert the current tendency to overlook it and raise the attention of the materials science community on the benefits of using oxygen functionalization in many state-of-the-art applications.

## 1 Introduction

Many carbon precursors have oxygen in their structure, and thus, the residual oxygen functionalities remain in the carbonaceous network even at high temperatures. This is especially valid when reduced graphene oxides or carbonaceous materials obtained from biomass are used. Oxygen is bigger and more polar than nitrogen. Thus, when oxygen is introduced in carbon, the doping results in a more significant decrease of the conductivity and greater disruption of the carbon sp^2^ network. At the same time, the neighbor carbon atoms may have even lower electron density than carbons adjacent to nitrogen heteroatoms. That makes them perfect sites for adsorption and catalysis. Thus, it is quite surprising that, while the effect of nitrogen doping in carbon materials is well-established, the effect of oxygen has been much less explored. Many reviews on heteroatom-doped carbons (with nitrogen, sulfur, and phosphor) do not discuss the effect of oxygen,^1−5^ and the studies that discuss oxygen functionalities are often ambiguous about the definition of the oxygen functional groups (OFGs). As a consequence, there is a lack of in-depth research on the role of oxygen atoms in terms of the properties of materials and the effect that they have in different applications.

It has already been shown that even as little as a few wt % of oxygen atoms (the amount a little bit higher than that of carbon blacks) can dramatically influence the properties of carbons. For instance, 4 wt % of oxygen atoms introduced on the surface of multiwalled carbon nanotubes (MWCNTs) made them work as OER electrocatalysts with a performance comparable to that of the state-of-the-art transition metal catalysts.^6^ Further series of experimental data and DFT calculation suggested that −COOH and C–O–C groups were particularly effective in, i.e., ORR, OER, and CO_2_RR.^7−9^ Thus, it is not surprising that OFGs have also been reported to significantly influence the performance of supercapacitors, batteries, and electrocatalysts beyond OER, even when they are present in relatively low amounts.^10−14^ Moreover, OFGs can bind cations serving as anchors for metal single sites and also facilitating adsorption of cations during electrochemical cycling processes having a positive effect on metal-ion batteries.^15^

Considering the (often not intentional) omnipresence of oxygen in carbon materials and its significant effect on catalytic and energy storage properties, it is clear that there is a need for a better understanding of the state of oxygen in doped carbons and their effect on (electro)catalytic properties. Oxygen groups also provide condensation pathways for the formation of cross-linked carbonaceous structures from molecular precursors and polymers. For instance, they activate attached carbon atoms toward nucleophilic substitution facilitating the extension of π-conjugated networks. In other words, they are key to the cross-linking ability of many precursors, and thus, some synthetic strategies could be used to produce model carbon materials to understand OFGs effects.

In this Mini-review, we discuss recent publications on the effect of oxygen in carbon material and its properties and performance in electrocatalytic and energy storage applications and stabilization of metal sites to raise the reader’s attention on considering OFGs as a valuable tool to prepare advanced functional materials. In the last section, we review a selected set of bottom-up approaches for the controlled synthesis of carbonaceous structures comprising tailored OFGs to inspire new research and discussion on this topic.

## 2 Elucidation of Oxygen Functional Groups

**Figure 1:**
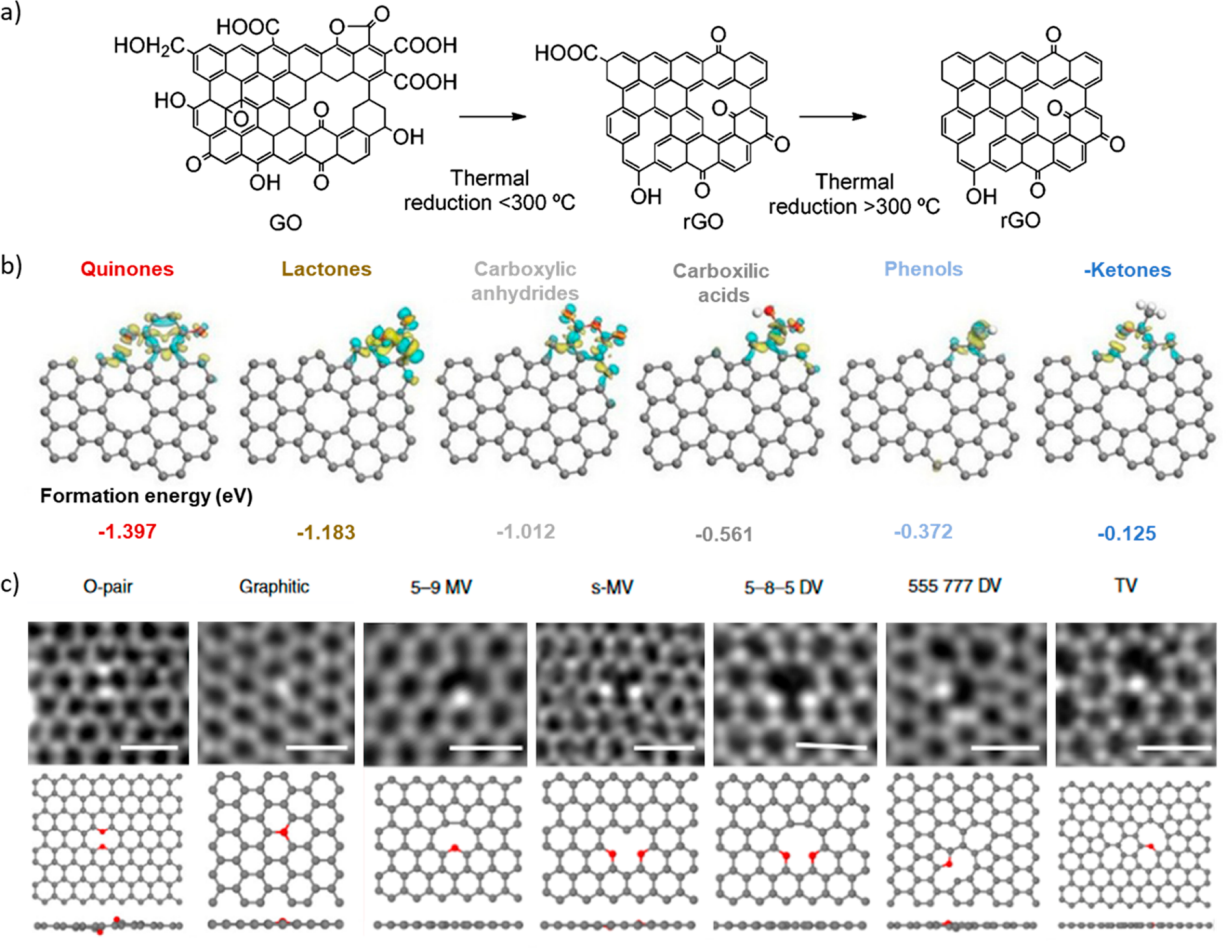
(a) Scheme of the transformation of graphene oxide into reduced graphene oxide as a representation of different OFGs and how they change upon thermal annealing. Reproduced from ref (16) with permission from the Royal Society of Chemistry. (b) Calculated formation energies of different oxygen functional groups. Adapted with permission from ref (17). Copyright 2021 John Wiley & Sons. (c) STEM images of different configurations of oxygen atoms in graphene. Adapted with permission from ref (19), Copyright 2019, The Author(s).

Oxygen atoms can exist in numerous states in carbon materials. They change in amount and type with different temperature treatments, as exemplified in Figure 1a using the thermal reduction of graphene oxides.^16^ Probably, the most common OFGs are hydroxyl, carboxylic, and epoxide. Hydroxyl and epoxide OFG can exist in the basal plane or in the edge of a graphitic carbon layer. When they are found in the basal plane, they disturb the network conjugation as often happens in graphene oxides. Carboxylic groups can be found on the edge of graphitic layers and can be considered as in-plane OFGs which can be conjugated to the sp^2^ network. Other in-plane functionalities are pyron, ether, carbonyl, and quinone groups (Figure 1a). Introducing in-plane oxygen in the basal planes of graphitic carbon is more challenging to achieve and control. In this context, the formation energies of several OFGs were studied by Li et al. to understand which ones will stabilize on the surface of defected carbon structures (Figure 1b).^17^ The studied OFGs were quinones, lactones, carboxylic anhydrides, carboxylic acids, phenols, and ketones. All of the calculated formation energies were negative, meaning all these groups can theoretically be stabilized on the surface of carbons. The formation energies demonstrate that quinone, lactone, and carboxylic anhydride OFGs are easier to form than carboxylic, phenol, or ketone groups. Experimentally, it has already been proven that different OFG can be inserted in the final network depending on the precursor, synthetic procedure, and activation or post-treatment to which carbon is submitted.^18^

OFGs have traditionally been associated with the aforementioned in-plane or basal plane functionalities. However, new electron microscopy studies revealed that oxygen can also be present in different substitutional sp^2^ lattice configurations in the form of stable ether-like bonds. Hofer and co-workers frequently observed configurations with two oxygen atoms substituting two adjacent carbon atoms or graphitic oxygen atoms making up to three bonds to adjacent atoms (Figure 1c). The functional properties of such doping remain unknown. Moreover, their results seem to point to the existence of stable oxygen atoms next to carbon vacancies able to survive several beam exposure cycles to capture different images.^19^ Overall, the structural features of the defects present in N- and O-doped carbons turned out to be very similar to differences lying mainly in bond lengths and stability. The unexpected existence of triple-bonded O further highlights the similarities of these two heteroatoms. These similarities can be seen as a potential advantage to tune O-doped performance for different applications, since surface oxidation can be achieved relatively easily. Regarding the triple-bonded oxygen atoms, they can be considered pyrylium groups in which oxygen atom carries a positive charge and participates in the electronic conjugation. Though these OFGs can exist in carbon structures, only a few reports discussed them. Interestingly, such oxygen configuration can provide new functionalities to carbonaceous structures such as redox switches or photosensitizers.^20^

## 3 Influence of Oxygen Functional Groups in Electrochemical Applications

Herein, it is important to highlight that most studies on carbon functional materials provide neither a clear definition of the state of oxygen in the material nor the role that the OFGs play in its physicochemical properties or performance in electrochemical applications. In this section, we will discuss the influence of different oxygen groups in different carbon materials on energy-related applications.

### OFG’s Effects on Supercapacitors

Even in carbons intentionally doped with other heteroatoms, OFGs are often present on the surface having a significant influence on the material’s electrochemical behavior. They can (1) enhance the wettability of the surface of carbons when using aqueous electrolytes and (2) work as redox-active functionalities. OFGs that undergo redox transformations add a certain faradaic contribution to the total specific capacitance. Though this usually works at the slight expense of the power density of the cell, increasing the amount of O can remarkably raise the cell’s volumetric and gravimetric capacitance and therefore serve as an important factor for improving the overall performance of supercapacitors.

**Figure 2:**
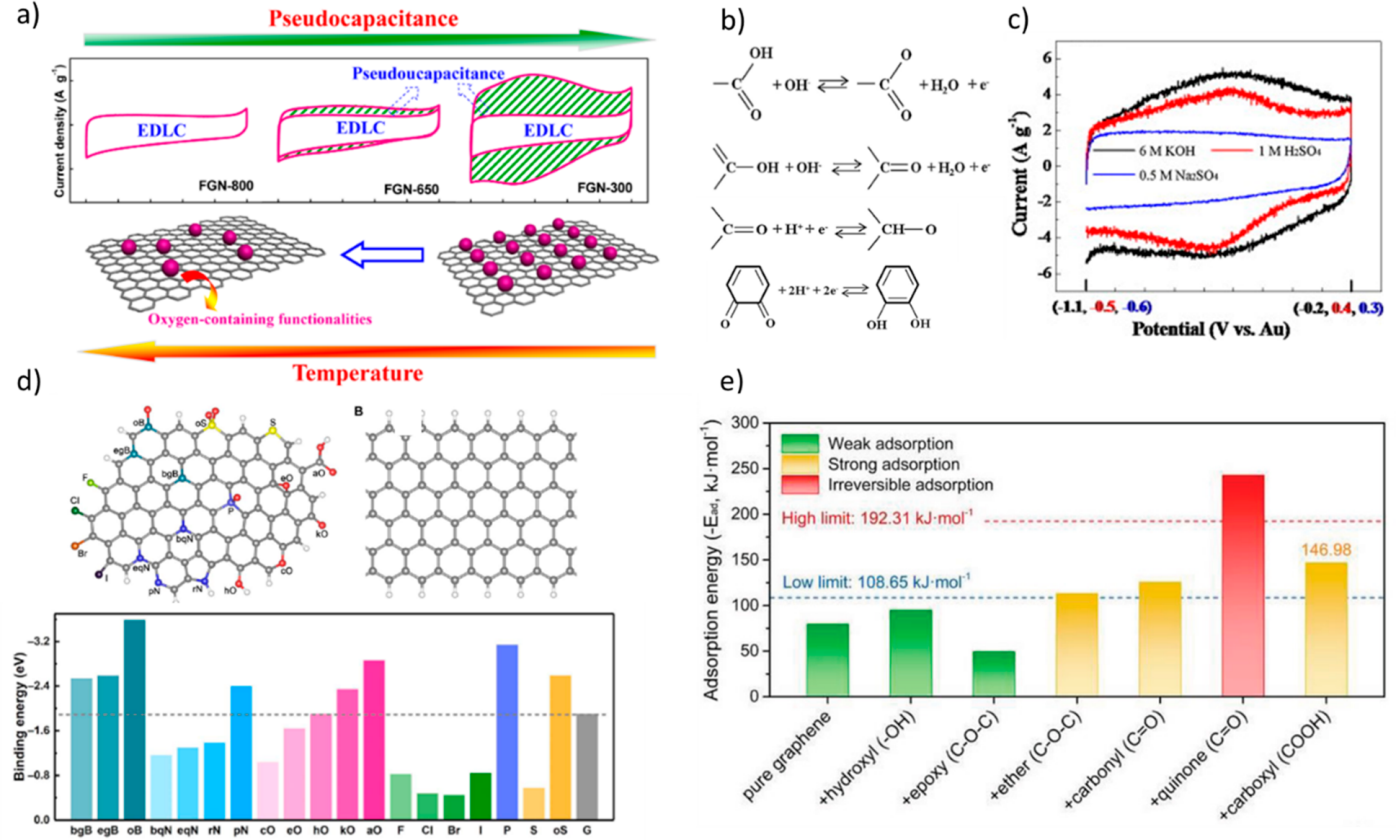
(a) Illustration of the relationship between thermal temperature and pseudocapacitance. Reproduced from ref (10). Copyright 2014 American Chemical Society. (b) Examples of redox active functionalities and (c) CV curves of RGO (200 °C) in three electrolytes at scan rate of 20 mV s^–1^. Adapted with permission from ref (21). Copyright 2013 Jong-Huy Kim. Published by Elsevier Ltd. (d) Summary of calculated binding energy between heteroatom-doped carbon and a Li atom. The hydrogen, boron, carbon, nitrogen, oxygen, fluorine, phosphorus, sulfur, chlorine, bromine, and iodine atoms are marked as white, green, gray, blue, red, cyan, violet, yellow, bottle green, orange, and black, respectively. Reproduced with permission from ref (15). Copyright 2019 The Authors, some rights reserved. (e) Adsorption energies of one Na atom over the carbon surfaces modified by typical oxygen-containing functional groups. Dashed lines represent the Na cohesive energy and adsorption energy corresponding to irreversible adsorption. Reproduced with permissions from ref (27). Copyright 2020 Wiley-VCH GmbH.

In 2014, Yan et al. prepared reduced graphene oxide via a very gentle thermal reduction of graphene oxide at 300 °C in the presence of Mg(OH)_2_. The resulting material had an O/C atom ratio of 0.2 and −C=O, −C–O, −C–OH, and −COOH OFGs at the surface. Increasing the temperature to 600 and 800 °C resulted in the decrease of the O/C atom ratio to 0.06 and a slight increase in the surface area of the samples (from 285 to 319 m^2^/g). Despite the increase of the conductivity of the samples prepared at higher temperatures and their slightly larger specific surface area, the sample prepared at 300 °C showed better performance when used as a supercapacitor electrode (Figure 2a). The authors ascribed the enormous volumetric energy density (27.2 Wh L^–1^) to the large faradaic contribution (pseudocapacitance) and improved interaction of the electrolyte with the oxygen-containing surface of the electrode material prepared at lower temperatures.^10^ Similarly, the pyrolysis of *Perilla frutescens* leaves yielded carbon materials with over 20 wt % of oxygen and volumetric energy density of 14.8 Wh L^–1^.^11^ The authors also ascribed the enhanced performance of the carbonaceous materials to the large heteroatom content promoting surface wettability and great pseudocapacitance.

OFGs acting as redox-active functional groups (Figure 2b) are very dependent on the electrolyte nature and pH. For instance, quinone and carbonyl groups are active in acidic media, while acid organic groups such as carboxylic, lactone, phenol, or lactol provide pseudocapacitance in basic media. The importance of the functional groups and pH was explored by Oh et al. using reduced graphene oxide electrodes. In neutral media, pseudocapacitance was not observed (Figure 2c).^21^ While the stability of acidic groups in alkaline electrolytes was very good, the pseudocapacity of basic groups in acidic media was significantly reduced. In organic electrolytes, oxygen groups were ascribed to reduce the reversibility and decrease the performance at higher current rates.^22^ Nevertheless, more efforts should be invested in clarifying which OFGs are stable under which conditions. It is also highly possible that the stability of these groups depends on the electrochemical conditions and the type of carbon they are attached to. For instance, You Ren Hu et al. prepared C–OH and C=O Kuraray carbon materials using a controlled electrochemical oxidation approach. The formation of the aforementioned groups suppressed the formation of −COOH with inferior conductivity. More importantly, the formed OFGs were very stable in acidic media upon 10,000 cycles.^23^

OFGs can also be introduced *in situ* during the cycling process in aqueous electrolyte especially when broad potential windows are applied. This might improve the performance during the cycling process.^23^ However, it is worth remembering that the presence of OFGs has also been reported to have negative effects. The most pronounced is the increased internal resistance of carbon. Moreover, bulky OFGs, such as −COOH, were associated with disruption of the electrochemical double layer (EDL) lowering the non-faradaic capacitance contributions.^24^

Another established strategy relies on doping carbon materials with redox-active molecules. One of the most typical strategies involves introducing quinone–hydroquinone reversible redox pairs through molecular impregnation. In this context, Illic et al. published the preparation of vanillin-decorated chitosan electrodes through ball milling. The resulting materials contained guaiacyl groups that, after demethylation, enabled quinone–hydroquinone reversible pairs on the surface of the electrodes. As a result, a large pseudocapacitive contribution was added to the materials electrochemical response as capacitors.^25^ This approach is especially interesting since it bridges the gap between biomass-derived precursors and carbons and specific OFG decoration. For instance, vanillin can be produced industrially from lignin, and, as demonstrated by Illic and co-workers, it can easily be used as a doping agent to enhance the capacity of carbon-based materials.

### OFGs Effects on Metal-Ion Batteries

Surface oxygen functionalities also improved the capacity and power density of carbon electrodes in Li-ion batteries. This is because functional groups on carbon can drive the uniform Li deposition without the formation of dendrites. In 2019, Chen et al. used first-principles calculations and experimental verifications to understand the lithiophilicity of heteroatom-doped carbon surfaces.^15^ The study was conducted using graphene nanoribbons single-doped and codoped with different heteroatoms (i.e., B, N, O, F, Cl, Br, I, P, and S). Interestingly, the highest affinity toward lithium was calculated for −COOH and −C=O functionalities. On the other hand, epoxy groups showed poor performance due to lithium interaction from the opposite site of the graphene plane (Figure 2d). Furthermore, experimental data confirmed that the lowest nucleation overpotential was found on O-doped carbon materials. In 2020, Cai et al. ran DFT studies to explain the enhanced performance of Li-ion capacitors based on quinone and ester modified carbon materials. The results pointed out the especially beneficial role of these groups in Li^+^ adsorption.^12^

The introduction of redox-active quinone functionalities through molecular doping of carbon materials was also used to enhance the performance of Li-ion batteries by Illic et al.^13^ The bisvanillonitrile (another derivative from lignin) was polymerized over carbon black to facilitate the contact between the current collector and the oxygen-rich polymer. The introduced quinone functionalities enhanced the overall performance of the materials as cathodes for Li-ion energy storage devices. Moreover, the introduction of the functional groups via polymerization of bisvanillonitrile over the carbon materials avoided the need of using additional binders.

Heteroatom doping has also been explored as a strategy to enhance Na^+^ adsorption in sodium-ion batteries. In 2018, Ghimbeu et al. showed that oxidation of carbon surfaces does not necessarily result in an enhanced performance. For instance, the oxidation of cellulose-derived carbons did not result in a higher battery capacity despite a 5-fold increase in the oxygen content.^14^ Theoretical and experimental data revealed that not every OFG has the same effect on the performance of Na-ion batteries. In 2020, the effect of different OFGs on the conductivity of mesoporous ordered carbons and their adsorption capabilities of Na^+^ was systematically studied by Hanquing Zhao et al.^26^ The study of the density of states for individual OFGs showed that carboxylic acids, ketones, and lactones may lower the intrinsic electronic conductivity of the carbons, whereas carboxylic anhydrides and quinones translate into good electronic conductivity. It was also found that these groups and lactones have the best affinity toward sodium adsorption. Later, in 2021, Sun et al. synthesized carboxyl-rich anthracite based carbon doped with up to 20.12 at% oxygen via a mechanochemical process. The introduced −COOH groups were found to act as active sites for Na^+^ capacitive adsorption mainly due to electrostatic interactions. As a result, the materials showed a very large reversible capacity of 382 mA g^–1^ at 30 mA g^–1^. Moreover, their presence facilitated diffusion controlled Na^+^ insertion due to *d*-spacing expansion. The authors also calculated the absorption energy of Na^+^ on different OFGs by DFT and indicated that there is an optimum energy window. The lower limit is the metallic cohesion energy of Na, and on the other hand, excessively high energy would cause irreversible adsorption as calculated for quinone (Figure 2e).^27^

The results described above point to the great benefit that introducing OFGs in carbon networks might have in the performance of batteries. Though some experimental and theoretical studies were carried out, more research efforts should be invested in further understanding the effect that each type of OFG has on the performance of the batteries. Moreover, the understanding of the effect of codoping in the materials also needs to be further studied. In this context, Xie et al. has very recently reported on the post-treatment of phosphate-treated carbons with a secondary carbonization to change the proportions between irreversible and reversible capacity in P/O heteroatom configurations for anode materials. While C–O and PO_3_^–^ were responsible for irreversible capacity, C=O and PO_2_^3–^/PO_4_^3–^ groups foster reversible capacity in P/O-codoped carbon anodes and their concentration could be enlarged by the post-treatment.^28^

### Electrocatalysis

In 2014, Li and Su laid out a first step to understand the (electro-)catalytic activity of different OFGs. According to their DFT calculations, quinone groups present the highest nucleophilicity of noncharged oxygen species (i.e., carboxyl, diketone, ketone, lactone, and quinone) on graphene.^29^ This points not only at quinone groups as very active toward electrophilic attack but also at the importance of loading one type of OFG over others for the final performance of an oxidized carbon. Later on, several papers were reported in which controlled oxidation of carbon and nanocarbon surfaces proved to have a remarkable effect on their electrocatalytic properties, especially toward the oxygen reduction reaction (ORR) and the oxygen evolution reaction (OER).

**Figure 3:**
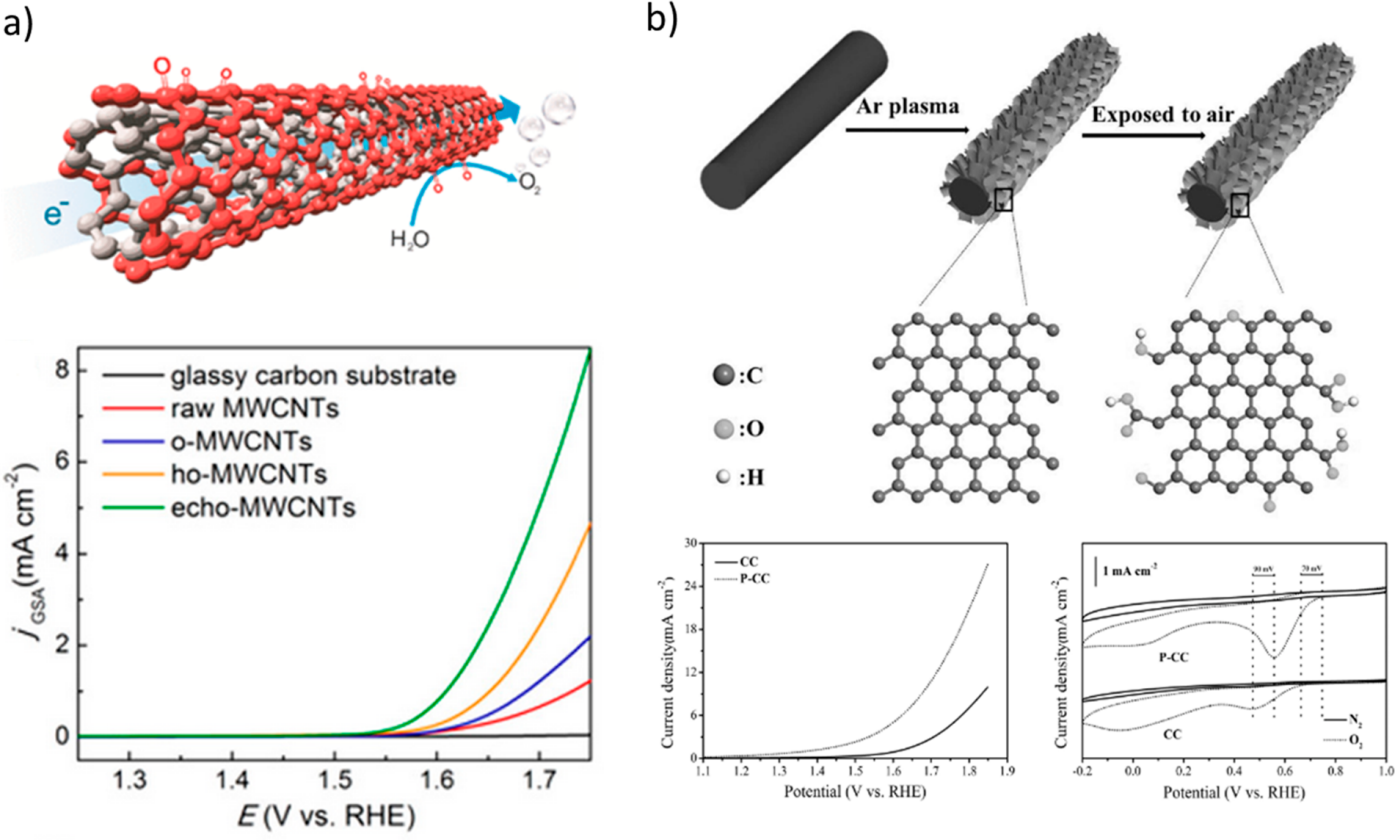
(a) Polarization curves obtained with MWCNTs of different treatment stages coated glassy carbon electrodes in 0.1 M KOH at a scan rate of 5 mV s^–1^. Reproduced from ref (6). Copyright 2015 American Chemical Society. (b) (Top panel) In situ exfoliation of edge-rich and oxygen-functionalized graphene from carbon fiber by submitting fibers to plasma etching and subsequent exposure to air and (bottom panel) performance comparison of the bare material and the treated material as electrocatalysts for the OER and ORR. Adapted with permission from ref (9). Copyright 2017 WILEY-VCH Verlag GmbH & Co. KGaA, Weinheim.

In 2015, Lu et al. performed the oxidation of MWCNTs in three consecutive steps: oxidation in piranha solution, hydrothermal treatment, and electrochemical oxidation. Each step significantly improved the OER electrocatalytic performance of the materials reaching the end the performance yielded by the state of the art transition metals (with oxygen contents up to 6 at%). Such OER activity was rationalized by the OFGs such as ketonic C=O, which altered the electronic distribution of the surrounding carbon atoms at the MWCNT surfaces and facilitated the adsorption of water oxidation intermediates. The improvement was observed even when the content of O was reduced by half after hydrothermal treatment (Figure 3a). This was attributed to removal of less stable OFG and increased conductivity of MWCNTs.^6^

Li et al. produced edge-rich and oxygen functionalized graphene (up to 9 at%) from carbon fibers by plasma etching and subsequent exposure to air. The resulting material catalyzed the ORR through the 4e^–^ transfer mechanism and shows a ca. 0.2 V improved overpotential for the OER (Figure 3b). The authors rationalized the results by the synergic effect of OFG and carbon defects introduced in the final material throughout the synthesis. Their supporting DFT studies revealed that the large decrease of the OER overpotential and the favorable four-electron-transfer mechanism for the ORR is caused by carboxylic groups next to carbon defects and carbonyl groups close to nondefective carbons.^9^

On the contrary, Lu et al. reported in 2018 an almost linear correlation between the ORR activity of oxidized carbon nanotubes (CNT) and the content of O (up to 9 wt %). Moreover, they validated their oxidation strategy using other carbon materials like carbon black. At the same time, the oxidation of the CNTs enhanced the activity and selectivity of the material toward H_2_O_2_ production (i.e., 2-electron-transfer mechanism) during the ORR. Their DFT studies assigned the high catalytic activity of the materials to −COOH at the armchair edged and C–O–C OFG in the basal planes of graphene lagers.^7^

Regarding the production of H_2_O_2_, it is worth remembering that, at an industrial scale, it is synthesized through the anthraquinone oxidation process. In this context, Han et al. showed experimentally and theoretically that indeed carbon enriched in quinone groups shows much higher selectivity toward H_2_O_2_ compared to other OFG (containing 10–20 at% of O). They introduced quinone groups by mechanochemical activation of graphitic nanoplates and further oxidation of broken bonds with gaseous oxygen. Although DFT calculations showed that the 1,4-quinone functionalities incorporated in the basal planes would have the highest activity, the introduction and stabilization of such groups is unlikely. High activity was also calculated for 1,2-quinones located at the edges. They are easily formed and therefore were assigned for high activity toward H_2_O_2_.^30^

The previously described reports show that relatively low content of O, on the order of a few weight percent, can dramatically enhance the performance during electrocatalysis. It has also been reported that electrochemical cycling, as well as chemical oxidation, can oxidize the carbon material’s surface and cause the enhancement of the material’s electrocatalytic behavior. At the same time, the high performance can be achieved only if the electronic conductivity is good, so the integrity of sp^2^ is preserved. This is the reason most of the reports cited above start from a predefined carbon material that is then oxidized in post-treatment. In such cases, oxygen atoms react with defective sites which are usually located at the graphitic edges, not disrupting the conjugation of the system, and thus, the conjugated structure is preserved.

### Stabilization of Metallic Active Sites for Electrocatalysis

The strong affinity of oxygen atoms toward transition metals makes OFGs perfect anchors to carbon structures. Currently, N-coordinated metals with a prominent porphyrin-type coordination M–N_4_ are almost exclusively single-atom catalyst examples on carbon surfaces. However, in comparison to nitrogen, oxygen has higher electronegativity and therefore can be efficiently used to further tune the electronic structure of metal catalytic active centers. Nevertheless, the reports on oxygen stabilized SAC are scarce.

Even if a metal center is coordinated by nitrogen, the close proximity of oxygen can have remarkable repercussions on its activity. DFT calculations showed that the electronic configuration of the Co–N_4_/C center is dramatically altered by the attachment of an oxygen atom to the adjacent carbon atoms. This resulted in a change in the ORR mechanism. For instance, the presence of one or two oxygen atoms changed the charge state of Co by 0.05 e^–1^ and 0.10 e^–1^, respectively. This increased the Δ*G*_OOH*_ making the cleavage of the O–O bond difficult and driving the ORR toward H_2_O_2_ production. Moreover, the results were confirmed experimentally showing that indeed the presence of oxygen (epoxy groups) next to Co–N_4_/C sites leads to the production of hydrogen peroxide.^31^

**Figure 4:**
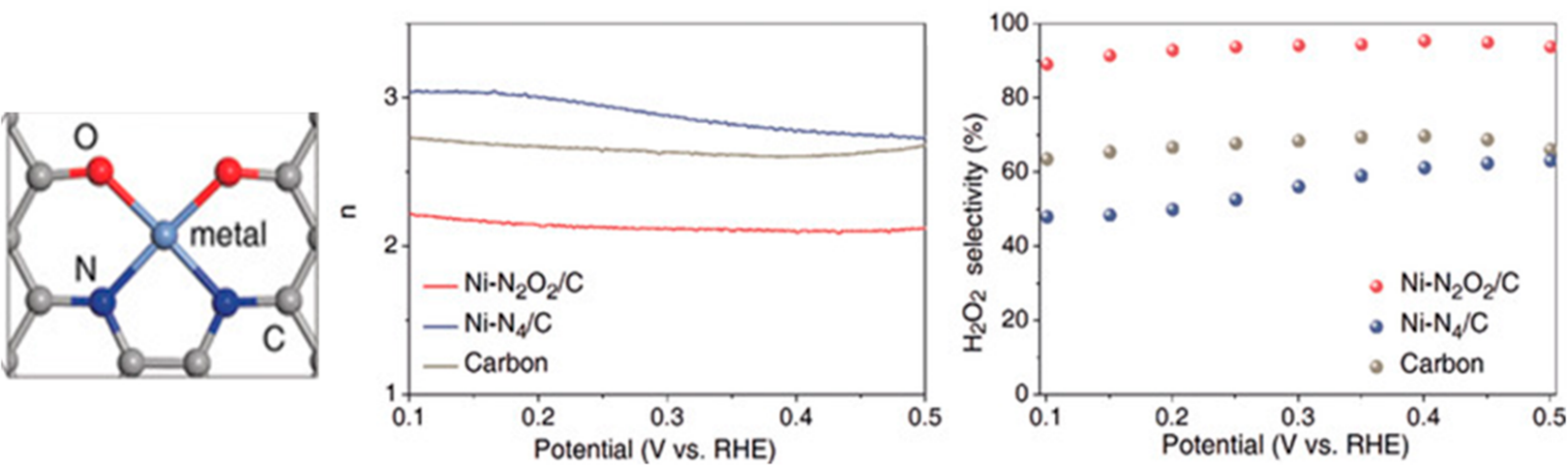
Calculated average number of electrons involved in ORR; H_2_O_2_ selectivity as a function of applied potential. Adapted with permission from ref (32). Copyright 2020 Wiley-VCH Verlag GmbH & Co. KGaA, Weinheim.

Carbon-supported Ni (II) single atoms with a Ni–N_2_O_2_/C tetradentate coordination were shown to be an efficient ORR catalyst toward H_2_O_2_ too. The catalyst was prepared by adsorption of a Ni (II) complex with a tetradentate Jacobsen’s ligand on a carbon black and subsequent pyrolysis at 300 °C in Ar. The catalyst achieved over 90% Faradaic efficiency (FE) toward H_2_O_2_. It showed high stability under alkaline conditions and at high current densities. On the other hand, carbon supported Ni single atoms with a Ni–N_4_/C coordination achieved a lower selectivity toward the ORR and showed an electron transfer number of 2.77 and 40–50% FE toward H_2_O_2_ (Figure 4). This study demonstrates the importance of the coordination of metal atoms, which greatly influences the mechanism of catalysis.^32^

In this context, Chen et al. prepared Fe_1_N_4_–O_1_ by fast pyrolysis of FeN_4_ ligands over an oxygen-rich carbonaceous support prepared using l-alanine. The iron is coordinated to 4 nitrogen atoms resilient from the porphyrin structure and an axial O from the support. A sample without O was also prepared for comparison. The samples were used as electrocatalysts for the electrochemical CO_2_ reduction reaction. The prepared Fe_1_N_4_–O_1_ achieved nearly 100% FE_CO_ over a wide potential range (−0.56 V to −0.87 V vs RHE). The result was explained by the axial O ligand inducing the Fe 3d orbit to shift to a lower energy level which results in a rapid CO desorption making the production of CO prevail over the evolution of hydrogen. In contrast, the sample prepared with an O poor support showed mainly selectivity toward hydrogen.^33^

Our group has recently reported on the effect of using oxygen coordinated metal ligands as a precursor for the production of metal single site or cluster electrocatalysts instead of metal porphyrins. We have prepared a series of highly nitrogen doped carbonaceous materials that are very resistant upon oxidation and that withstand the decomposition of metal acetates or acetylacetonates at mild temperatures (300–400 °C). Following this strategy, we fabricated electrocatalysts consisting of Cu^II^/Cu^I^ clusters (up to 4 wt % of Cu) homogeneously dispersed on the surface of a nitrogen-doped carbonaceous precursor derived from the carbonization of an ionic liquid in salt melts. The oxygen coordination of copper facilitated oxygen binding during the ORR and promoted a fast 4-electron transfer mechanism.^34,35^

## 4 Bottom-up Synthesis of Oxygen-Containing Carbons

In the last section, we discuss the importance of different functional groups on the performance of carbon-based materials in different electrochemical applications. However, the rational design of carbon materials with heteroatoms sitting in defined positions and in defined states is challenging, no matter the chosen heteroatom. In the specific case of oxygen doping, the material design is more challenging than, for example, nitrogen-doped carbons, since the removal of oxygen heteroatoms is easier at higher temperatures.^36^ In fact, some oxygen functionalities (e.g., carboxylic groups) are less prone to be stabilized during the heating process and usually must be introduced during post-treatment. In this section, we describe a selection of bottom-up strategies to introduce oxygen functional groups in carbon-based materials ranging from well-known phenol–formaldehyde polycondensations to carbon suboxide polymeric derivatives with the aim to inspire new materials chemists searching for new strategies to prepare oxygen-functionalized carbons.

### Phenol–Formaldehyde Strategy

**Figure 5:**
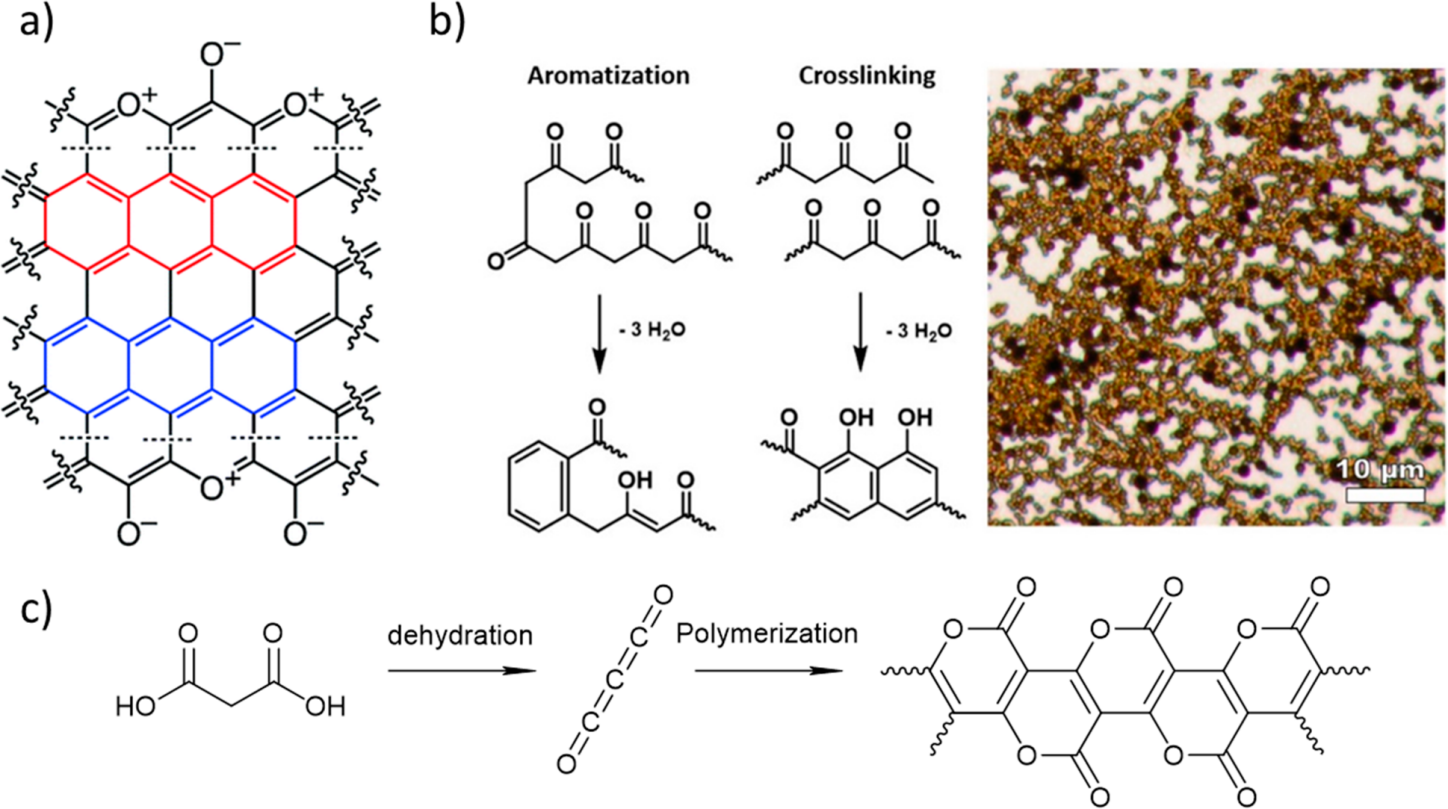
(a) Idealized fused aromatic core in carbons derived from the pyrolysis of resorcinol, phenol, or phluoroglucinol polycondensation resins at 800 °C. Highlights in red and blue are different aromatic repeated layers. On the edges, there are pyrilium functional groups. Reproduced from ref (37) with permission from the Royal Society of Chemistry. (b) Scheme of aromatization and cross-linking of polyketo chains and optical microscopy picture of acetic anhydride derived carbonaceous products obtained by hydrothermal condensation at 250 °C. Adapted from ref (45). Copyright 2021 American Chemical Society. (c) Carbon suboxide synthesis scheme.

In this context, the classical phenol–formaldehyde strategy to prepare carbons through heat treatment of cross-linked resins should not be overlooked. For instance, in 2017, Far and co-workers published a comprehensive study on the chemical transformations during pyrolytic conversion of phenolic resins to carbons.^37^ In their study, they analyzed the evolution of the chemical structure and functional groups of phenol–formaldehyde, resorcinol–formaldehyde, and phloroglucinol–formaldehyde resins, as well as a phloroglucinol–terephthalaldehyde upon heat treatment. The samples were gelled using an HCl-catalyzed synthesis and dried in supercritical CO_2_ yielding monolithic aerogels. Then, they were submitted to a thermal treatment at 240 °C in air atmosphere. This first step caused ring-fusion aromatization with the formation of a skeletal carbon backbone formed by fused pyrylium heteroaromatic rings of phloroglucinol derived resins (Figure 5a). On the other hand, phenol and resorcinol resins underwent an oxidation process in which −CH_2_– bridges were transformed into C=O moieties. Interestingly, after treating all samples at 600 °C under Ar, they converged to a similar composition. Moreover, further carbonization at 800 °C produced pyrylium groups in every sample regardless the original composition of the resin. The presence of pyrylium groups in all these “mainstream” carbons might seem a bit exceptional, but Lawrinenko and Laird also reported in 2015 the presence of such OFG in biochars from carbonized corn and cellulose. Interestingly, the presence of these groups was assigned to provide significant anion exchange capacities to the carbon materials, which highlight, once again, the importance of understanding the oxygen functionalities not only from synthetic but also from natural precursors.^38^

The oxidation pretreatment that Far and co-workers performed over phenolic resins also affected the porous network obtained after carbonization of the samples. The carbons that experienced a ring-fusion aromatization step exhibited larger pore volumes after carbonization. The authors claimed that the increased surface area of the samples was beneficial for gas sequestration and ion-exchange technologies.^37^ Later, in 2021, controlled preoxidation of phenol–formaldehyde resins prior to carbonization was also reported to have a direct effect on the final accessible surface area of derived hard carbon materials. Using a longer preoxidation time, lower specific surface area hard carbons were obtained which increased up to a 22.2% the reversible capacity of the materials when used as anodes in sodium ion batteries. As a result, the materials reached capacities of up to 334.3 mAh g^–1^ at 20 mA g^–1^. Moreover, the hard carbons contained larger amounts of carbonyl groups which increased their degree of disorder and acted as retention sites for Na^+^ adsorption.^39^

Herold et al. tried recently to establish analytical standards to analyze OFGs by temperature-programmed desorption, diffuse reflectance infrared Fourier transform spectroscopy (DRIFTS), and potentiometric titration. To do so, they prepared phluoroglucinol–formaldehyde resins and derived carbons at 850 °C and subsequently oxidized their surface using HNO_3_ at atmospheric pressure and under hydrothermal conditions. While the pristine carbons had an oxygen content of 2.6 wt %, the HNO_3_ oxidized ones contained up to 31.9 wt %. Interestingly, the authors also run a selective defunctionalization of the samples via LiAlH_4_ reduction and protective group chemistry strategies to tune the OFGs on the surface of the oxidized carbons.^40^ As a result of the LiAlH_4_ reduction, mainly hydroxyl groups remained in the carbon surface and were utilized for chemical grafting. By using the protective group strategy, −C=O groups were preserved during the reduction, and quinones, ketones, and aldehyde groups could be obtained. Not only does the study show a good strategy to generate specific functional groups on carbon-based materials, but also Herold and co-workers stablished that the “desorption” of −OH groups from amorphous carbons occurs at temperatures from 400 to 550 °C.

### Hyper-Cross-Linked Polymer Strategy

Hyper-cross-linked polymers (HCPs) differ substantially from classical polymers of the same chemical nature. Their polymer network is extremely interconnected making the backbone very rigid which, in certain conditions, fosters the formation of intrinsic porosity. In 2002, Davankov postulated basic principles for synthesizing hyper-cross-linked polymers, which can be applied to a broad range of monomers.^41^ The resulting highly porous HCPs are proven to be useful as a base for a new generation of adsorption materials.^41^ Since then, the research on HCPs has experienced rapid growth due to their remarkable advantages such as diverse synthetic conditions, easy functionalization, and postsynthetic treatments. These offer possibilities for the construction of well-defined porous cross-linked polymer networks with customized micromorphology and functionalities.^42^ Thus, HCPs are a suitable precursor for the synthesis of porous carbonaceous structures, in which the well-defined functional groups allow for control of heteroatom doping in the resulting carbonaceous materials.

In 2017, HCPs were synthesized by Zhang et al. using a Friedel–Crafts alkylation reaction of phenyltrimethylsilane and formaldehyde dimethyl acetal as a cross-linker. The HCP was subsequently carbonized at 600, 700, and 800 °C using potassium hydroxide as the activating agent. The oxygen content of the samples decreased with increasing carbonization temperature and the main OFGs present in each sample were C–O, C=O, and O–C=O. The carbonization resulted in at least a 2-fold increase of surface area (compared to HCP) reaching the maximum of 3101 m^2^ g^–1^ at 700 °C. The large surface was encouraging for energy storage applications like in supercapacitors or in Li-ion batteries. The sample prepared at 600 °C showed the best performance with a discharge capacity of 1220 mA h^–1^ at 100 mA g^–1^ in a Li-ion battery. It also achieved the highest capacitance of 379 F g^–1^ in a supercapacitor setup at 0.5 A g^–1^ with 91.2% capacitance retention after 3000 cycles at 2 A g^–1^. Many previous studies have shown that disordered porous carbons can exhibit large Li storage due to the presence of defects, heteroatoms, and cavities. In this set of samples, there is no clear trend between capacity and porosity, and the best performing sample has the lowest surface area. Therefore, the high reversible charge capacity was attributed to oxygen which is higher than for samples prepared at 700 and 800 °C. The same reasoning was applied to the best performance in the supercapacitor setup.^43^

Also in 2017, Xu and co-workers reported the preparation of dual cross-linked polydivinylbenzene (PVDB) tubes via cationic polymerization of divinylbenzene and secondary Friedel–Crafts cross-linking. The resulting carboxylic acid functionalized PVDB tubes were pyrolyzed at 850, 900, and 950 °C for 2 h in nitrogen atmosphere. The obtained carbons were doped with oxygen and retained the tubular, bamboo-like morphology of the original HCP. Again, the main OFGs were COOH, C=O, and C–O, and the sample carbonized at 900 °C contained up to 6.85 at% of oxygen. The samples had porous networks comprising micro-, meso-, and macropores and surface areas ranging from 445 to 610 m^2^/g. The carbons were tested as electrodes in aqueous supercapacitors. The one carbonized at 900 °C exhibited high volumetric capacitance, 254 F cm^–3^, moderate volumetric energy density, 12.9 Wh L^–1^ at 428 W L^–1^, and excellent cycling stability, with capacitance retention of 96.9% after 10,000 cycles at 428 W L^–1^. Such high capacity and excellent stability were ascribed to the presence of OFG at the surface which improved the wettability in aqueous electrolyte and provided additional capacity through faradaic processes. The relatively high operating voltage of 1.4 V was also attributed to the positive influence of O functionalities.^44^

### Keten Condensation Strategy

Besides the well-known polycondensation reactions to generate phenolic resins and the HCPs, acetic anhydride self-polymerization can be used to produce oxygen-rich carbonaceous materials. This strategy was recently explored by Rat et al.^45^ who studied the self-polymerization of acetic anhydride and acetic anhydride mixed with l-histidine. Under hydrothermal conditions at mild temperatures (up to 250 °C), polyketene chains form from acetic anhydride self-condensation. The chains undergo subsequent cross-linking through dehydration reactions, ultimately forming aromatized substituted phenyl rings. During this dehydration-driven process, about a third of the original oxygen contained in the polyketene chains is eliminated as water. The final material contains ca. 20% of oxygen in the form of C=O and C–O–C epoxide OFG. The copolymerization of acetic anhydride with l-histidine is described to achieve additional functionalization of the carbonaceous materials. The resulting oxygen- and nitrogen-rich materials contain not only acid but also basic sites and thus become catalytically active toward acetal hydrolysis and subsequent Knoevenagel condensation reaction as well as CO_2_ cycloaddition on epoxides.^45^ On the other hand, the pure acetic anhydride derived material only showed conversion to catalyze the acetal hydrolysis.

One remarkable outcome from the work by Rat and co-workers is the very large surface area that the final condensation products (with or without the comonomer) achieved at the very mild temperatures (ca. 250 °C). They exhibit large microporous volume and specific surface areas ranging from ca. 300 (for pure acetic anhydride derived materials) to up to 1000 m^2^ g^–1^ for the materials prepared with l-histidine. Though not well described in the manuscript, one can hypothesize that the release of up to two-thirds of the original oxygen content of the samples should be responsible for such high porosity at such mild conditions.

### Carbon Suboxide Condensation Strategy

Carbon suboxide is a highly reactive member of the carbon oxides family with a linear O=C=C=C=O structure. This molecule undergoes self-polymerization reaction at temperatures below 0 °C generating highly conjugated structures as indicated by their intensive dark coloration. The synthesis of carbon suboxide was described for the first time in the 19th century by Brodi,^46^ who observed the formation of a dark red solid which he referred as “red carbon” during the electric decomposition of carbonic-acid gas. Nevertheless, apart from several research papers during the 20th century, the polymer did not receive broad attention. At the current state, the structure of the polymer is described as a ribbon made of fused 2-pyrone units (Figure 5c). It means that at very low temperatures we can access a conjugated ladder polymer with lactone functionalities located at the edges of the ribbons. This is perhaps one of the most elegant and facile synthetic approaches for rigid polymeric structures. In fact, the polymer can be seen as *cis*-polyacetylene limited by −O–C=O. This is by no means a trivial issue, since it might increase the processability of polyacetylenes.

The chemistry of carbon suboxide and the polymer is largely forgotten; however, in our group we have recently developed a new facile synthesis and investigated the semiconducting properties of the polymer.^47^ Poly(carbon suboxide) is an extremely versatile platform which can be used as such (as organic semiconductor) and perhaps serve for the synthesis of low-temperatures carbonaceous structures with defined chemical composition. For instance, it has already been shown that the polymer can thermally decompose through pure decarboxylation releasing only CO_2_ or a mixture of CO_2_ and CO.^48^ This can produce different but well-controlled OFG in carbonaceous structures. It also might lead to different model structures for in-depth studies of functional properties which would help in rational design of carbons. We believe that there is a bright future for carbon suboxide polymer as both organic semiconductor and precursors of low temperature carbonaceous structures.

## 5 Conclusion and Outlook

The collection of papers summarized here highlights something that should come as no surprise for any materials chemist: OFGs dramatically change the performance of carbon-based materials in a wide range of (electrochemical) applications. This was maybe explored more when using the materials as carbocatalysts; however, the chemical functionalization with redox active groups, surface polarity changes, and affinity to a number of molecules and ions including those of common electrolytes or conductivity changes, among many other effects, alters their performance beyond pure catalysis. Despite the severe effects on the physicochemical properties of the carbon materials, the characterization of these OFGs is still challenging, and their descriptions in papers are usually vague or ignored. We hope this Mini-review will encourage researchers to pay more attention to the characterization of OFGs.

The surprising stability of unconnected neighboring oxygen atoms in the carbon lattice that stand several cycles during STEM imaging or even the presence of oxygen bound to three carbon atoms (i.e., graphitic oxygen potentially behaving similar to graphitic nitrogen) must serve as a driving force to further systematically analyze oxidized carbon surfaces using new and more advanced techniques. For instance, such functional groups pave the way to look for very reactive functionalities similar to carbenes. Moreover, the systematic study of model samples with other techniques like TPD or DRIFTS can also help with understanding the OFGs present in different materials at different temperatures in a much more accessible way. This strategy can be very powerful when combined with other spectroscopic techniques.

The usage of oxygen-doped carbons in electrochemical state-of-the-art applications needs controlled and “on demand” specific oxygen functional groups. There is a plethora of possibilities to pre-code specific OFGs in carbon precursors from well-established synthesis to quite innovative ones. The thorough analysis of carbons prepared using the well-established polycondensation of phenols and aldehydes disclosed the existence of pyrilium functional groups on the surface of all samples, and Friedel-Crafts cross-linking fostered the formation of oxygen-rich hyper-cross-linked polymers and oxygen-doped and porous derived carbons. Also, some promising new condensation routes based on the use of acetic anhydride to prepare oxygen-rich functional carbon materials are still basically unexplored. Beyond the bottom-up strategies showcased here, some a posteriori routes also were mentioned.

Furthermore, given the changes in selectivity and activity when even one or two oxygen atoms are in the coordinating environment of single site electrocatalysts, it should be of special interest for materials chemists to explore the metal ligands beyond the Me–N_4_ species as single atom precursors. In this context, it is worth pointing out that, though the systematic study of OFGs is crucial, the synergy with other heteroatoms should not be overlooked. For instance, Me–N_2_O_2_ single sites already behaved electrochemically different.

The potential of grafting of redox-active molecules on the surface of carbon materials is a simple method of preparing model electrocatalysts to evaluate the influence of specific OFGs in any electrochemical application as well as to evaluate the validity of characterization techniques on analyzing the nature of oxygen functionalities. Along the same line, the use of controlled reduction using specific protecting groups to vary the proportion of different OFG is without any doubt a strategy that should be further explored.

Despite the many advances in the field of carbon materials for several (electrochemical) applications achieved over the past decades, there is still plenty of room to understand certain fundamental aspects of their physicochemical nature. We hope this work serves as an inspiration for many researchers to have a second look especially at their material’s oxygen functionalities and evaluate the impact they have on the performance for any give application.
